# T32. USING PSYCHOSOCIAL INTERVENTION TO ENHANCE KNOWLEDGE OF ATTENUATED PSYCHOSIS SYMPTOMS AND HELP SEEKING BEHAVIORS AMONG AFRICAN AMERICAN YOUNG ADULTS

**DOI:** 10.1093/schbul/sby016.308

**Published:** 2018-04-01

**Authors:** Huijun Li, Tanisha Pelham

**Affiliations:** 1 Florida A&M University; 2 Florida State Hospital

## Abstract

**Background:**

The lack in knowledge of mental illnesses is of primary concern with regard to help-seeking and treatment outcomes, especially when faced with chronic and severe illnesses such as psychotic disorders. Where mental health knowledge lacks, so does the ability to recognize the signs and symptoms, risk factors, and causes of mental disorders; as well as the appropriate routes of care for these illnesses. Psychotic disorders and attenuated/subclinical psychotic symptoms are often the target of stigma due to the distinctive symptoms, disruptive behavior and perceived dangerousness of both. Furthermore, the social stigma and discrimination historically faced by African Americans in the United States magnifies the disparity in treatment outcomes among this population. The enrollment of minority college students has increased from 15 percent to 33 percent over the past three decades; cases of students with mental illnesses have also increased. It is becoming more important to explore psychosocial intervention strategies geared to promote knowledge of attenuated psychotic symptoms and help-seeking behavior among African Americans young adults.

**Methods:**

The sample consists of 177 students from a Historically Black College and University (HBCU) in the Southeast region of the United States. The participants ranged in age from 18–25. A within group test-retest design was used to conduct the study. The participants received a pretest, participated in a psychosocial training on attenuated psychosis syndrome, and a posttest.

**Results:**

The results suggest that the training was effective in enhancing the participants’ knowledge of early warning signs of psychosis and improving their help-seeking behavior. However, stigma unexpectedly increased after the training.

**Discussion:**

Discussion: Enhancing mental health literacy has implications for influencing the effects of stigma and discrimination. Colleges and universities are optimal settings for improving mental health literacy because of the high-risk age groups served at these institutions. Mental health literacy is an important life skill that should be taught before the need arises. In order to increase the likelihood that African American college students seek the appropriate help for mental health problems and understand the effects of stigma on help seeking behaviors, more cultural specific interventions are necessary among this population. Interventions should include strategies to cope with stigma and discrimination in order to reduce the effects of both. Future research in this area should also consider how one’s ethnic identity correlates with stigma and help seeking behavior.

